# Analysis of Differential Alternative Splicing in Largemouth Bass After High Temperature Exposure

**DOI:** 10.3390/ani14203005

**Published:** 2024-10-17

**Authors:** Xianxian Zhao, Yizhou Wang, Zhenlu Wang, Tianma Luo, Jun Huang, Jian Shao

**Affiliations:** 1 College of Animal Science, Guizhou University, Guiyang 550025, China; 18486302954@163.com (X.Z.); wangzl@gzu.edu.cn (Z.W.); 18786109114@163.com (T.L.); shaojian5098@163.com (J.S.); 2 Key Laboratory of Animal Diseases and Veterinary Public Health in Guizhou Province, Guiyang 550025, China; 3 Hubei Fisheries Science Research Institute, Wuhan 430077, China; 13697335706@163.com

**Keywords:** largemouth bass, environmental stress, transcriptome sequencing, differential alternative splicing

## Abstract

Temperature is one of the critical factors affecting the physiological functions of fish. With ongoing global warming, changes in water temperature have a profound impact on fish species. Alternative splicing, being a significant mechanism for gene expression regulation, facilitates fish to adapt and thrive in dynamic and varied aquatic environments. Our study used transcriptome sequencing to analyze alternative splicing in largemouth bass gills at 34 °C for 24 h. The findings indicated an increase in both alternative splicing events and alternative splicing genes after high temperature treatment. Specifically, the comparative analysis revealed a total of 674 differential alternative splicing events and 517 differential alternative splicing genes. Enrichment analysis of differential alternative splicing genes revealed significant associations with various gene ontology (GO) terms and KEGG pathways, particularly in immune-related pathways like necroptosis, apoptosis, and the C-type lectin receptor signaling pathway. These results emphasize that some RNA splicing-related genes are involved in the response of largemouth bass to high temperatures.

## Simple Summary

Altering aquatic environments with global warming can have a profound impact on fish species. This study employed transcriptome sequencing to analyze alternative splicing in the gills of largemouth bass after heat stress. We observed an increase in alternative splicing events (674) and differential alternative splicing genes (517). Enrichment analysis revealed significant associations with immune-related gene ontology (GO) terms and KEGG pathways, notably necroptosis, apoptosis, and C-type lectin receptor signaling. These results emphasize that some RNA splicing-related genes are involved in the response of largemouth bass to high temperatures.

## 1. Introduction

Temperature represents a crucial environmental factor essential for the survival and growth of fish, closely intertwined with their physiological processes [1]. Fluctuations in water temperature not only impinge upon growth rates and reproductive capacities but also heighten the propensity for disease outbreaks. Empirical evidence indicates that temperature fluctuations impact the prevalence of fish diseases, elevating the risk and susceptibility to bacterial infections and aggravating the severity of viral diseases [2,3]. With the persistence of global warming [4,5], characterized by extreme climatic events, rising mean temperatures, and heightened temperature variability, fish physiology is anticipated to confront significant challenges [6,7,8]. Particularly, extremely hot weather in summer can cause massive fish mortality [9]. Temperature changes pose a potential threat to aquaculture, which bear profound implications for the stable development and economic returns of aquaculture.

In recent years, with the advancements of RNA-Seq technology and evolution of various bioinformatics tools [10,11], transcriptome analysis has become more accessible and affordable within the realm of biological research. This paradigm shift offers a novel avenue for delving into the functionalities of various species. Concurrently, the unveiling of an increasing volume of fish genome data has provided a rich repository for investigating alternative splicing, which makes alternative splicing a hot research topic [12,13]. Alternative splicing is a phenomenon occurring during the transcription process, wherein a single gene is capable of generating multiple splicing variants, each exhibiting distinct expression patterns and functions within cells, tissues, or individuals. It induces a high complexity of transcriptomes [14]. The importance of alternative splicing lies in its ability to confer diverse biological functions by generating multiple protein isoforms without an increase in the total number of genes. Consequently, through alternative splicing, fish can express specific proteins at varying developmental stages, tissue types, or environmental conditions.

Alternative splicing, which allows a single gene to produce multiple proteins, is also prevalent among fish [15]. This trait not only increases the diversity of proteins and phenotypic characteristics [16], but also plays a crucial role in facilitating the adaptation of fish to various environmental changes and survival challenges. Research has indicated that environmental variables have been identified as significant determinants impacting alternative splicing in aquatic organisms. For instance, various environmental factors, such as bacteria [17], viruses [18,19], parasites [20,21], high temperature [22], low temperature [23], nanoparticles [24], and salinity [25,26], have been shown to influence the alternative splicing patterns of genes in fish. Moreover, in recent years, the application of alternative splicing extends across various research domains, including the development of subunit vaccines [27], the study of albinism-associated genes in the Wels catfish (*Silurus glanis*) [28], and the study of sex determination and gonadal differentiation [29,30]. The study of fish alternative splicing not only helps us to understand the law of life activities of fish, but also provides an important theoretical basis for fish breeding and disease prevention and control. Delving deeper into the mechanisms of alternative splicing in fish augments our understanding of their growth, developmental trajectories, environmental adaptability, and disease resistance. Such insights are crucial for bolstering the sustainable development of the fish farming.

Largemouth bass (*Micropterus salmoides*), also known as the California bass, is one of the most economically important fish species for freshwater aquaculture. It is favored by consumers for its palatable flavor and nutritional benefits. However, largemouth bass suffer from temperature challenge [31], and as the global demand for fish escalates, aquaculture’s role in ensuring food security becomes increasingly crucial [32]. Therefore, it is extremely necessary to better understand the response of largemouth bass to changes in high temperature stress. As an important functional organ in fish, gills are involved in many physiological processes [33,34]. Compared to other organs, the gill tissue is in direct contact with the water environment and is more susceptible to changes in water temperature than other organs. Studies of gill tissue help us better understand the effects of high temperatures on largemouth bass. In this study, we utilized the largemouth bass as a model organism to investigate alternative splicing genes in the gills using transcriptome sequencing technology. Our findings provide new insights into the regulatory of gene expression via alternative splicing in response to temperature fluctuations in largemouth bass.

## 2. Materials and Methods

### 2.1. Experimental Design

Largemouth bass were procured from a fish hatchery in Guangxi Province and were fed commercial fish feed twice daily for two weeks. Fish of uniform size and health were selected for the experiment, with an average body mass of approximately 35 ± 5 g. The experiment was divided into the control group and the high-temperature treatment group, each with three replicates of 20 fish with each replicate in a 300 L tank. The high-temperature treatment group (HTG) was gradually heated from 26 °C to 34 °C at a rate of 1 °C per day using a digital heating rod, and maintained at 34 °C for 24 h. Meanwhile, fish in the control group (CG) were maintained at 26 °C until sampling. Fish were fed normally during periods of elevated water temperatures. Subsequently, the fish were then euthanized with an overdose of MS-222, and gill tissues were collected. The collected tissues were immediately snap-frozen in liquid nitrogen and stored at −80 °C for subsequent RNA extraction.

### 2.2. Library Preparation and Sequencing

Total RNA was extracted using the TRlzol Reagent (Life Technologies, Carlsbad, CA, USA) according the instructions provided by the manufacturer. The concentration and purity of the RNA were quantified using NanoDrop 2000 (Thermo Fisher Scientific, Wilmington, DE, USA). The integrity of the RNA was assessed using the RNA Nano 6000 Assay Kit of the Agilent Bioanalyzer 2100 system (Agilent Technologies, Santa Clara, CA, USA).

After validating the samples, library construction was performed. Sequencing libraries were generated using Hieff NGS Ultima Dual-mode mRNA Library Prep Kit for Illumina (Yeasen Biotechnology (Shanghai) Co., Ltd., Shanghai, China). The quality of the libraries was subsequently evaluated using an Agilent Bioanalyzer 2100 system. Upon passing the library quality control, paired-end 150 (PE150) mode sequencing was performed using the Illumina NovaSeq6000 sequencing platform (Illumina, San Diego, CA, USA).

After filtering out reads containing low quality sequences, the clean reads were aligned to the reference genome using HISAT (2 2.0.4) software [35] to obtain the read alignment information. Subsequently, the mapped reads were assembled using StringTie [36] to reconstruct the transcriptome for further analysis, including differential expression analysis, gene function annotation, splicing event quantification, and function enrichment. Differentially expressed genes were identified using DESeq2 (1.30.1) software [37], with criteria set at |Fold Change| ≥ 2 and a false discovery rate (FDR < 0.01) for screening.

### 2.3. Alternative Splicing Event Quantification

The alternative splicing patterns were determined using ASprofile (b-1.0.4) software [38], which can categorize alternative splicing events into 12 types. This facilitated a comprehensive understanding of the various splice types. These types include alternative exon ends (AE), intron retention (IR), multi-intron retention (MIR), multi-exon skipping (MSKIP), skipped exon (SKIP), alternative 5′ first exon (TSS), alternative 3′ last exon (TTS), approximate alternative exon ends (XAE), approximate intron retention (XIR), approximate multi-intron retention (XMIR), approximate multi-exon skipping (XMSKIP), and approximate skipped exon (XSKIP).

### 2.4. Differential Alternative Splicing Events

We used the rMATS (4.0.2) software [39] to detect and quantify differential alternative splicing, setting a threshold of 5% for between-group difference in exon inclusion levels (|Δψ| > 5%) and applying a false discovery rate (FDR) with an adjusted *p*-value of <5%. The rMATS software can identify the five types of differential alternative splicing events, including skipped exon (SE), alternative 5′ splice site (A5SS), alternative 3′ splice site (A3SS), mutually exclusive exons (MXE), and retained intron (RI).

### 2.5. Enrichment Analysis

To further investigate the biological functionalities of differentially expressed genes (DEGs) and differential alternative splicing (DAS) genes observed in both control and high-temperature treatment groups, we performed gene ontology (GO) and the Kyoto Encyclopedia of Genes and Genomes (KEGG) enrichment analysis using the BMKCloud platform (www.biocloud.net) (accessed on 23 January 2024). The GO enrichment analysis of DEGs and DAS genes was implemented by the clusterProfiler [40], which is based on the Wallenius non-central hyper-geometric distribution. Additionally, the Kyoto Encyclopedia of Genes and Genomes (KEGG) database, a comprehensive resource for understanding high-level functions and utilities of biological systems generated by genome sequencing and other high-throughput experimental technologies, was utilized. These analyses facilitated the acquisition of insights into the functional roles and biological significance of these genes, thereby offering valuable information concerning the underlying molecular mechanisms associated with the observed alterations in gene expression.

### 2.6. Validation of Quantitative Real-Time PCR (RT-qPCR)

After sequencing, genes were selected for quantitative PCR (qPCR) validation, with primer sequences detailed in Table 1. We used the Thermo Fisher DNase I kit to remove genomic DNA. Complementary DNA (cDNA) was synthesized using a reverse transcription kit (Thermo Scientific, Waltham, MA, USA) using total RNA from the same batch as sequencing. Amplification primers were designed utilizing Primer5 (5.0) software and resources available on the NCBI website. The internal reference gene was β-actin and the relative gene expression was calculated by the 2^−ΔΔCT^ method.

## 3. Results

### 3.1. Statistic of Transcriptome Sequencing

To elucidate the effects of high temperature stress on the gills of largemouth bass, the transcriptomic profile of gills was investigated by RNA-seq. Table 2 provides an overview of the read counts and quality filtering metrics for the six libraries. After filtering low quality sequences, there were an average of 42,927,078 clean reads in the control group and 40,593,753 clean reads in the high temperature treatment group. The Q30 base ratio ranged from 96.45% to 97.47%, with an average GC content of 46.23%. In the control groups and high temperature treatment groups, the valid reads accounting 89.61% and 91.96% of the clean sequences were mapped to the genome, respectively. In the two groups, the percentage of unique clean sequences mapped to the genome was between 85.80% and 89.43%.

### 3.2. DEGs Analysis

**Figure 1:**
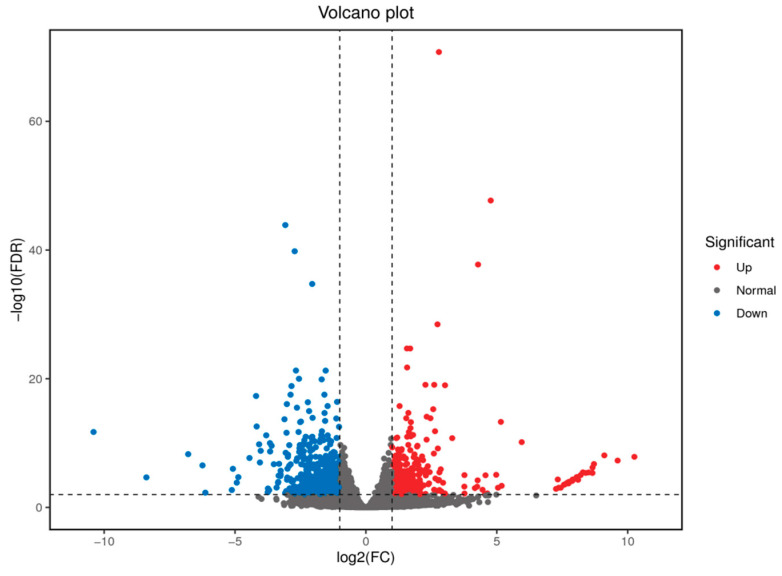
Volcano plot of DEGs statistics between the control and high temperature treatment groups. Red and blue dots represent up-regulated and down-regulated genes, respectively.

Gene expression analysis identified a total of 987 differentially expressed genes (DEGs) between the control and high-temperature treatment groups (Figure 1 and Table S1). Among these DEGs, 396 were up-regulated, while 591 were down-regulated, suggested that largemouth bass employ varied response strategies to cope with high temperatures. Notably, heat-shock related protein genes including hspa4a, hspa9, hspa5, hspbp1, hsp90b1, dnajb1a, dnajb2, and dnajc3b were significantly up-regulated (Table 3).

### 3.3. Alternative Splicing Profiles

**Figure 2:**
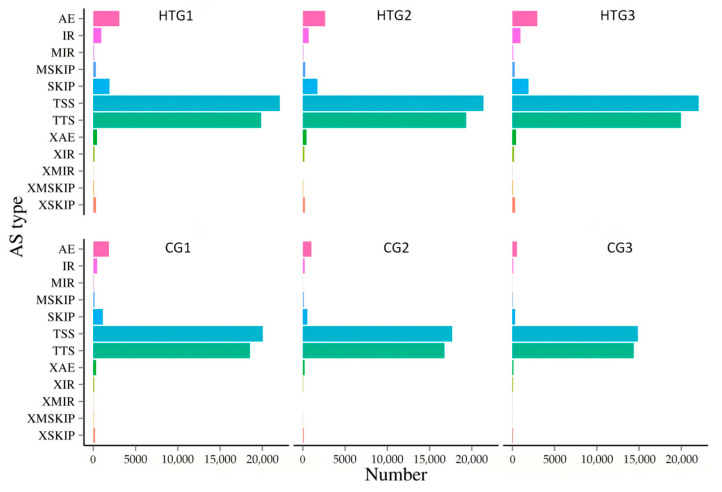
Statistics of alternative splicing events in the control and high temperature treatment groups. The horizontal axis is the number of variable shears under that type of event and the vertical axis is the classification of variable shear events. (1) AE: alternative exon ends (5′, 3′, or both); (2) IR: intron retention; (3) MIR: multi-IR; (4) MSKIP: multi-exon SKIP; (5) SKIP: skipped exon; (6) TSS: alternative 5′ first exon (transcription start site); (7) TTS: alternative 3′ last exon (transcription terminal site); (8) XAE: approximate AE; (9) XIR: approximate IR; (10) XMIR: approximate MIR; (11) XMSKIP: approximate MSKIP; (12) XSKIP: approximate SKIP. Different colors represent different splicing events.

Utilizing ASprofile software, twelve distinct types of alternative splicing (AS) were identified, including AE, IR, MIR, MSKIP, SKIP, TSS, TTS, XAE, XIR, XMIR, XMSKIP, and XSKIP. The distribution of these AS types is detailed in Table S2. Notably, compared to the control, high temperature treatment group significantly increased AS event types. Among these, TSS-types events were the most prevalent, followed by TTS, AE, and SKIP, with XMIR events showing the least prevalence. Sequencing data shown that a total average of 36,708 AS events across 15,664 genes in the control group, and 48,460 AS events across 18,510 genes in the high-temperature treatment group (Figure 2 and Table 4). Compared to the control group, the high temperature treatment group exhibited an increasing in alternative splicing events. These results indicated that largemouth bass exhibits a more pronounced occurrence of alternative splicing after exposure to a high-temperature environment. Largemouth bass can respond to temperature fluctuations by regulating AS events or changing splicing patterns.

### 3.4. Differential Alternative Splicing Analysis

**Figure 3:**
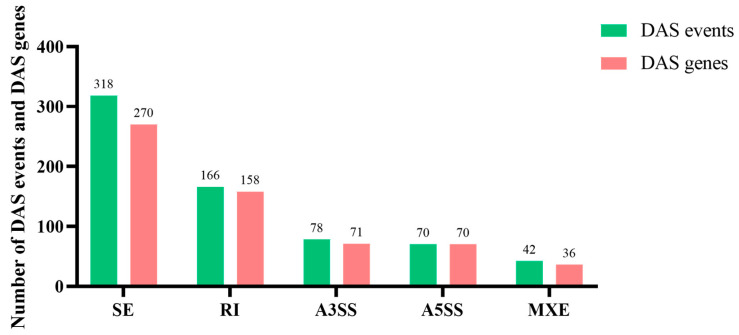
Number statistics of differential alternative splicing events and differential alternative splicing genes between the control and high temperature treatment groups.

Alternative splicing analysis was performed by the software rMATS, a statistical method for the robust and flexible detection of differential alternative splicing from replicate RNA-Seq data. In a comparison of these two groups, a total of 674 differential alternative splicing events and 605 differential alternative splicing genes are identified in these five splice types (Figure 3). Excluding one gene with multiple splice types, there are 517 differential alternative splicing genes (Table S3). Among the five types of DAS events, SE exhibited the highest enrichment, with a total of 318 events. This was followed by the RI event, involving 166 events. The A3SS was observed in 78 events, while the A5SS was detected in 70 events. Lastly, the MXE event was identified in 42 events.

### 3.5. GO and KEGG Analysis of Differential Alternative Splicing Genes

In the present study, a total of 674 differential alternative splicing events and 517 differential alternative splicing genes were identified. Thus, we hypothesized that these differential alternative splicing genes might be associated with high temperature stress. To further determine the biological functions of DAS genes occurring in the control group and the high temperature treatment group, differential alternative splicing genes were extracted for GO functional enrichment analysis and KEGG pathway enrichment analysis.

**Figure 4:**
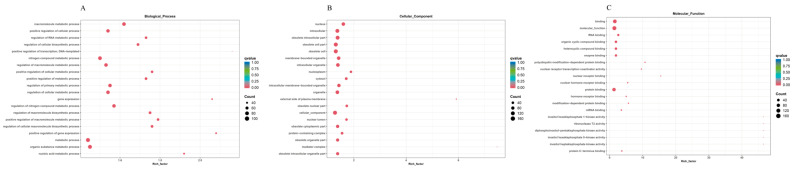
GO enrichment analysis of differential alternative splicing genes in the control and high temperature treatment groups. The top 20 category terms of differential alternative splicing genes were enrich in the biological process (**A**), cellular component (**B**), and molecular function categories (**C**).

Through the gene annotation results, the classification statistics of DAS genes at the secondary classification level of the GO database can visualize the main relevant functional entries of DAS genes (Figure 4A–C). In the biological processes, DAS genes were enriched in macromolecule metabolic process, positive regulation of cellular process, regulation of RNA metabolic process, regulation of cellular biosynthetic process, and positive regulation of transcription DNA-templated. Within the cellular component domain, the DAS genes were observed in the nucleus, intracellular, obsolete intracellular part, obsolete cell part, obsolete cell, membrane-bounded organelle, intracellular organelle, nucleoplasm, and cytosol. As for molecular functions, DAS genes were mainly enriched in many enzyme activities and binding function, such as RNA binding, organic cyclic compound binding, heterocyclic compound binding, enzyme binding, nuclear receptor binding, nuclear hormone receptor binding, protein binding, hormone receptor binding, and mRNA binding. These DAS genes were mainly related macromolecule biosynthetic and metabolic process, cellular metabolic process, gene expression, nucleus, intracellular, obsolete cell part, various enzyme activities, and various binding function. This indicates that high temperatures cause an intense reaction in the gill tissue cells of largemouth bass. This may be related to the cellular stress produced by largemouth bass in response to high temperatures.

**Figure 5:**
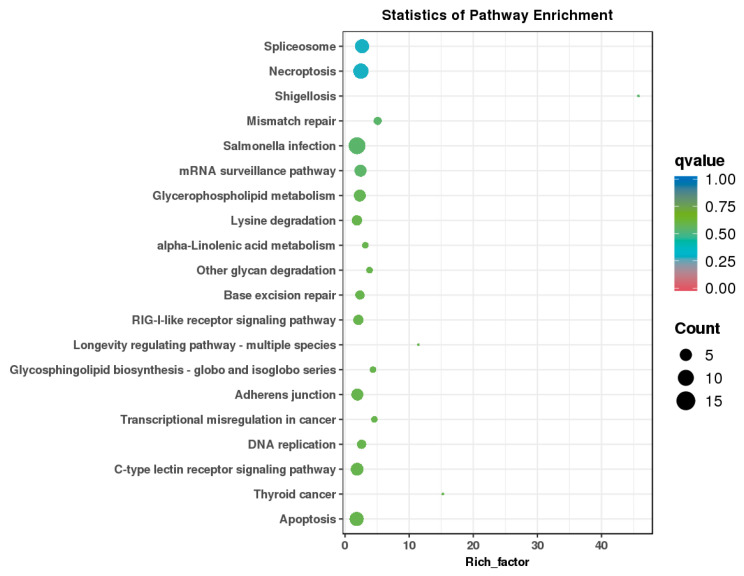
KEGG enrichment pathways of DAS genes.

To further investigate the potential biological pathways of largemouth bass after high-temperature treatment, we performed a KEGG enrichment analysis of the DAS genes occurring in the control group and the high temperature treatment group (Figure 5). The top 10 KEGG pathways enriched for DAS genes were necroptosis, spliceosome, salmonella infection, mismatch repair, shigellosis, mRNA surveillance pathway, glycerophospholipid metabolism, apoptosis, thyroid cancer, C-type lectin receptor signaling pathway, but not significantly. Notably, in these DAS genes, splice factors including *srsf10a*, *tra2b*, *u2af2a*, *dhx16*, *srsf7a*, and transcription factors including *myef2* were enriched in the spliceosome pathways.

### 3.6. Overlapping Genes between Differentially Expressed Genes and Differential Alternative Splicing Genes

**Figure 6:**
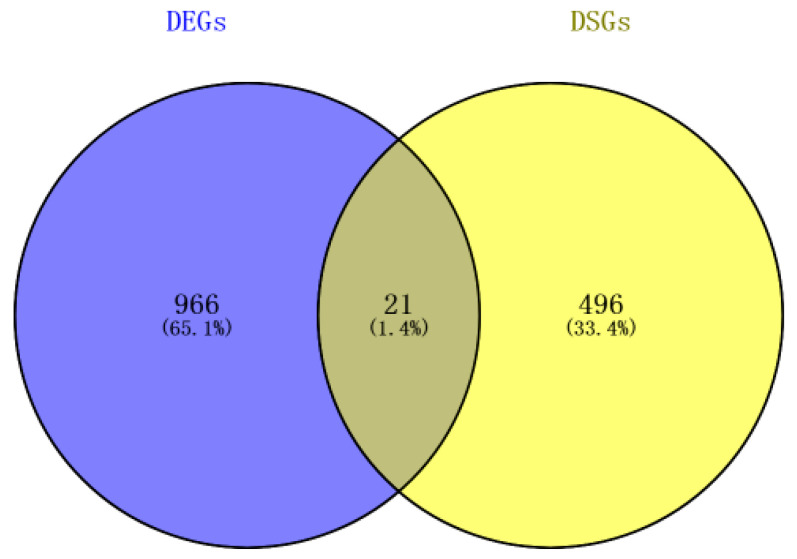
Genes presented between differentially expressed genes and differentially spliced genes.

In the comparative analysis between the control and high-temperature treatment groups, 987 differentially expressed genes (DEGs) and 517 differential alternative splicing genes (differentially spliced genes, DSGs) were identified. Additionally, 21 genes were found to be overlapping genes corresponding to 1.4% in total genes (Figure 6 and Table 5). Notably, heat shock protein genes including *hspbp1* and *dnajb2* are both differentially expressed genes and differentially spliced genes.

### 3.7. The atp2a1 Gene Analysis and RT-qPCR Validation

**Figure 7:**
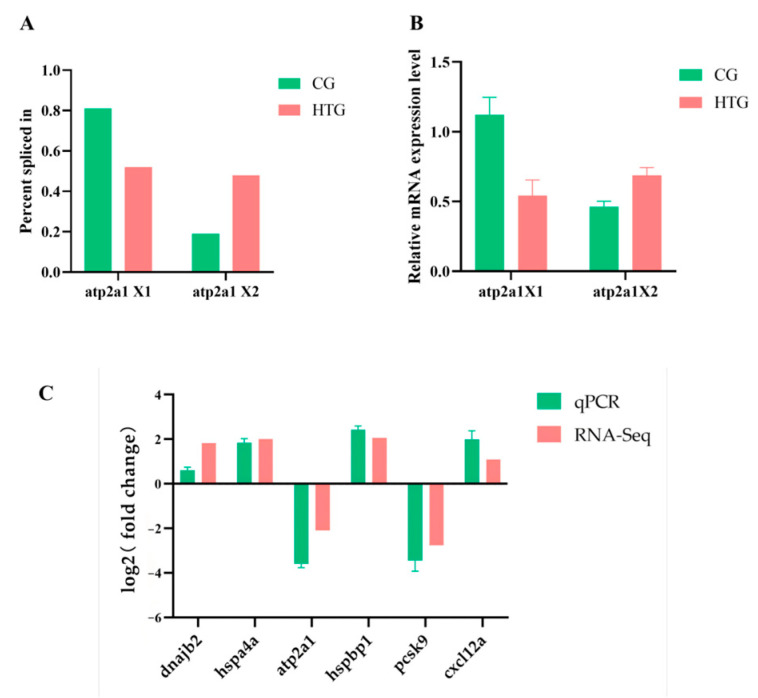
The *atp2a1* gene analysis and RT-qPCR validation. (**A**) Percent spliced in of *atp2a1*X1 and *atp2a1*X2. (**B**) Relative mRNA expression level of *atp2a1*X1 and *atp2a1*X2. (**C**) RT-qPCR validation of DEGs.

The overlapping genes *atp2a1* is a representative example that exhibits both significant differential alternative splicing and differential expression in the comparison groups. The *atp2a1* gene can generate two transcript isoforms in largemouth bass, namely isoform X1 (XM_038696950.1) and isoform X2 (XM_038696943.1). In this study, the type of differential alternative splicing event for the *atp2a1* gene is SE. In the control group, the level of exon inclusion is 19%, whereas in the high temperature treatment group, it is 48%. Similarly, the *atp2a1* gene was significantly down-regulated in DEGs. These alterations were further confirmed by RT-qPCR results (Figure 7A–C). The reliability of the RNA-seq results was verified using RT-qPCR. The results showed consistent trends of up- and down-regulated genes between RT-qPCR and RNA-seq (Figure 7C).

## 4. Discussion

Fish, as ectothermic organisms, are profoundly influenced by the temperature of the aquatic environment. The gills for fish can perceive the changes of temperature, salinity, pressure, and oxygen content in the water, so as to facilitate appropriate physiological and behavioral responses [41,42,43,44]. Studying gill tissue organs in fish can better provide us with an understanding of fish responses to environmental change.

Our results showed a total of 987 DEGs between the control and the high temperature treatment groups, with 396 DEGs being up-regulated and 591 DEGs being down-regulated. Compared to the control group, an increase in the number of alternative splicing genes and events was observed following the high temperature treatment. The results of our study are similar to those of previous studies. Previous studies have documented that heat stress induces an increase in alternative splicing in rainbow trout [22], catfish [45], grape [46], and rat [47]. Meanwhile, in the studies on rainbow trout and catfish, the most DAS events were in SE, similar to the present study. Furthermore, in addition to heat stress, both abiotic and biotic stressors, such as viral or bacterial infections and low salinity conditions, have been reported to trigger alternative splicing events in the pacific oyster and may be related to stress adaptation [48]. Our study demonstrates that exposure to elevated temperature in largemouth bass induces a distinct profile of differentially expressed genes and alternative splicing events.

In this study, the top 20 GO entries for biological processes, cellular components, and molecular functions were predominantly enriched in categories related to macromolecule biosynthetic and metabolic process, cellular metabolic process, gene expression, nucleus, intracellular, obsolete cell part, various enzyme activities, and various binding functions. Similarly, there are similar GO entries, such as binding, catalytic activity, cell part, and organelle in studies of the hybrid Jinhu grouper (*Epinephelus fuscoguttatus ♀ × Epinephelus tukula ♂*) and *Epinephelus fuscoguttatus*, and of yellow drum regarding temperature [49,50]. This suggests that high temperatures can trigger a reaction in cells. Additionally, it is noteworthy that the differential alternative splicing genes were enriched in immune-related pathways, including necroptosis, apoptosis, and the C-type lectin receptor signaling pathway, among others. Similarly, low temperature stress leads to enrichment of genes involved in the apoptosis, necroptosis, and the C-type lectin receptor signaling pathway in zebrafish [51,52]. Temperatures can affect fish immunity [53,54]. We speculate that alternatively splicing genes involved in immune-related pathways regulate the immune response in largemouth bass by producing a variety of transcripts.

Heat shock proteins (HSPs), also known as stress proteins and extrinsic chaperones, protect the cell from damage, play a significant role in adaptation to temperature [55,56], and are essential for maintaining cellular homeostasis [57]. Hsp90, a complex molecular chaperone, undergoes substantial conformational changes during the ATPase cycle [58]. Proteins of the Hsp90 family play important roles in cellular processes such as cell survival, apoptosis, immune responses, and hormone signaling, while they are critical for cellular responses and homeostasis under stressors [49]. Study found that hsp90 levels were higher in fish exposed to increased water temperatures compared to normal river temperatures. In our study, heat shock protein including *hspa4a*, *hspa9*, *hspa5*, *hspbp1*, *hsp90b1*, *dnajb1a*, *dnajb2*, and *dnajc3b* were significantly up-regulated. This indicates that elevated temperatures affects the expression of heat shock protein genes. Furthermore, in addition to temperature stress, virus and drug stress can alter the expression level of heat shock protein [59,60,61]. Notably, in this study, heat shock protein genes, including *hspbp1* and *dnajb2*, were identified as both differentially expressed genes and differentially alternative splicing genes. Temperature plays a crucial role in influencing both differential gene expression and gene splicing. Intriguingly, for DAS genes, splice factors such as *srsf10a*, *tra2b*, *u2af2a*, *dhx16*, and *srsf7a*, as well as transcription factors including *myef2*, were enriched in the spliceosome pathways.

Previous studies has demonstrated that the *atp2a1* gene plays a role in the inefficient calcium cycling pathway associated with heat production in red muscle, wherein the energy released from ATP catabolism is utilized for heat generation rather than for the transmembrane cycling of calcium ions [62]. In this study, we observed that the expression level of the *atp2a1* gene was lower in the high temperature treatment group compared to the control group. These results suggest that fish in a high temperature environment do not require the active generation of heat to maintain their body temperature. Consequently, the expression level of the *atp2a1* gene decreases in the high temperature treatment group. In addition, the qPCR results were consistent with the trends observed in the transcriptome data, thereby confirming the reliability of the transcriptome data.

In this study, we investigated alternative splicing in largemouth bass after high-temperature treatment by high-throughput sequencing. Of course, more in-depth studies are needed to fully understand the more detailed regulatory mechanisms and responses of alternative splicing in largemouth bass after high temperature.

## 5. Conclusions

This study identified 987 differentially expressed genes and observed increased alternative splicing events and AS gene occurrences in largemouth bass gills exposed to high temperatures. The alternative splicing genes were enriched in immunity-related pathways. These results suggest that high temperatures can have an effect on alternative splicing in largemouth bass.

## Figures and Tables

**Table 1:** Primers used for qPCR of genes.

Gene Description	Gene Symbol	Primers For RT-qPCR(5′-3′)
actin, beta 2	*actb2*	F:AAAGGGAAATCGTGCGTGAC R:AAGGAAGGCTGGAAGAGGG
HSPA (heat shock 70 kDa) binding protein, cytoplasmic cochaperone 1	*hspbp1*	F:GTTTCTGTGTTTATAGGGGGAGT R:CAGCCACATTTTCCTCTCCTCT
heat shock protein 4a	*hspa4a*	F:AGGAGGGACTGAGTGATTGTG R:TATAGCGGCGTTGGGGTAAG
DnaJ heat shock protein family (Hsp40) member B1a	*dnajb1a*	F:GATCGCTTTGGAGAAGAAGGATT R:AACATTGCGTGAGGGTCTCC
DnaJ heat shock protein family (Hsp40) member B2	*dnajb2*	F:GCAAACGCACCACTACCAAG R:TCTGGCTCCACAGGTGATCT
RAB38b, member of RAS oncogene family	*rab38b*	F:TTGAGACCTCCGCTAAGGACA R:TCGAGGTGAGGTGAGATGGT
proprotein convertase subtilisin/kexin type 9	*pcsk9*	F:TACAGGCCACCCAATGATGG R:TGAGCACTCGTCCTTCAACC
ATPase sarcoplasmic/endoplasmic reticulum Ca2+ transporting 1	*atp2a1*	F:GAACGCCATTGTCAGAAGCC R:CTGACCAAGGGAAACACCGT
sarcoplasmic/endoplasmic reticulum calcium ATPase 1 isoform X1	*atp2a1X1*	F:CACATACCTGGAGGGGAAAGTC R:ACTTTTGGCAGCTCTCTCTGG
sarcoplasmic/endoplasmic reticulum calcium ATPase 1 isoform X2	*atp2a1X2*	F:CCCCGTAACAAAACAAGGGAAAGT R:CTCAGCCTTTTCTCTCCACCC

**Table 2:** Summary of sequencing data.

SampleID	Total Reads	Mapped Reads	Uniq Mapped Reads	Multiple Map Reads	GC (%)	Q30 (%)
CG1	47,993,594	43,266,911 (90.15%)	41,671,714 (86.83%)	1,595,197 (3.32%)	45.95	96.45
CG2	41,734,410	37,580,439 (90.05%)	36,284,239 (86.94%)	1,296,200 (3.11%)	45.16	97.15
CG3	39,053,230	34,620,287 (88.65%)	33,507,306 (85.80%)	1,112,981 (2.85%)	44.89	97.25
HTG1	41,336,990	38,343,069 (92.76%)	36,871,254 (89.20%)	1,471,815 (3.56%)	46.93	96.84
HTG2	40,990,434	36,972,059 (90.20%)	35,481,860 (86.56%)	1,490,199 (3.64%)	47.47	97.47
HTG3	39,453,836	36,670,123 (92.94%)	35,284,978 (89.43%)	1,385,145 (3.51%)	47.01	97.43

**Table 3:** Heat-shock-related protein genes.

Gene_Name	nr_Symbol	log2FC	Regulated
EVM0025100	hspa4a	2.0	up
EVM0004810	hspa9	1.3	up
EVM0006180	hspa5	2.0	up
EVM0019730	hspbp1	2.1	up
EVM0020972	hsp90b1	1.6	up
EVM0002211	dnajc27	−1.3	down
EVM0000478	dnajb1a	1.7	up
EVM0008502	dnajb2	1.8	up
EVM0006974	dnajc3b	1.5	up

**Table 4:** Number of 12 types alternative splicing events.

Types	CG1	CG2	CG3	HTG1	HTG2	HTG3
TSS	20,041	17,655	14,841	22,053	21,364	22,032
TTS	18,535	16,756	14,359	19,848	19,333	19,944
AE	1859	1030	543	3078	2639	2958
SKIP	1115	523	313	1901	1703	1889
IR	445	220	119	940	686	974
XAE	316	194	127	440	417	434
XSKIP	188	116	78	316	239	322
MSKIP	152	105	48	310	283	279
XIR	115	56	79	152	169	185
MIR	54	31	10	102	78	112
XMSKIP	36	27	11	55	42	47
XMIR	11	7	9	28	15	12

**Table 5:** Overlapping genes between differentially expressed genes and differential alternative splicing genes.

Gene_Name	nr_Symbol	log2FC	Regulated	IncLevelDifference
EVM0005564	*phospho1*	−1.549253246	down	−0.107
EVM0007969	*g3bp2*	1.110357019	up	0.085
EVM0003895	*LOC115575854*	−1.043066212	down	0.088
EVM0007693	*LOC104925498*	−1.306175217	down	0.252
EVM0008366	*rfc4*	1.413957195	up	0.059
EVM0022841	*osbpl1a*	1.1280337	up	−0.365
EVM0011633	*LOC116067203*	−1.189173132	down	0.165
EVM0019730	*hspbp1*	2.054830224	up	−0.41
EVM0006360	*echdc2*	1.734738546	up	−0.059
EVM0016326	*rbm47*	1.152743561	up	0.141
EVM0001998	*atp2a1*	−2.090757937	down	−0.286
EVM0004218	*LOC111574170*	−2.176020596	down	−0.141
EVM0020426	*sra1*	1.473394669	up	0.075
EVM0008502	*dnajb2*	1.817428245	up	0.62
EVM0024754	*LOC117257900*	−1.058045498	down	0.117
EVM0007573	*col6a3*	−1.461908505	down	−0.117
EVM0017239	*elna*	−1.583695653	down	0.366
EVM0022857	*LOC118331090*	−1.339277604	down	0.477
EVM0000844	*vdac3*	−1.098536139	down	0.417
EVM0001431	*cnpy1*	1.4915122	up	−0.249
EVM0005617	*LOC116036998*	−1.698127263	down	−0.208

## Data Availability

The raw RNA-seq reads are available in the NCBI SRA (BioProject ID: PRJNA1107482).

## Supplementary Materials

The following supporting information can be downloaded at: https://www.mdpi.com/article/10.3390/ani14203005/s1, Table S1: “CG_vs_HTG DEGs”. Table S2: CG_vs_HTG alternative splicing types stats. Table S3: CG_vs_HTG DASgenes.
